# A Two-Level, Intramutant Spectrum Haplotype Profile of Hepatitis C Virus Revealed by Self-Organized Maps

**DOI:** 10.1128/Spectrum.01459-21

**Published:** 2021-11-10

**Authors:** Soledad Delgado, Celia Perales, Carlos García-Crespo, María Eugenia Soria, Isabel Gallego, Ana Isabel de Ávila, Brenda Martínez-González, Lucía Vázquez-Sirvent, Cecilio López-Galíndez, Federico Morán, Esteban Domingo

**Affiliations:** a Departamento de Sistemas Informáticos, Escuela Técnica Superior de Ingeniería de Sistemas Informáticos (ETSISI), Universidad Politécnica de Madrid, Madrid, Spain; b Department of Clinical Microbiology, Instituto de Investigación Sanitaria-Fundación Jiménez Díaz University Hospital, Universidad Autónoma de Madrid (IIS-FJD), Madrid, Spain; c Centro de Biología Molecular “Severo Ochoa” (CSIC-UAM), Consejo Superior de Investigaciones Científicas (CSIC), Madrid, Spain; d Centro de Investigación Biomédica en Red de Enfermedades Hepáticas y Digestivas (CIBERehd), Instituto de Salud Carlos III, Madrid, Spain; e Unidad de Virología Molecular, Laboratorio de Referencia e Investigación en Retrovirus, Centro Nacional de Microbiología, Instituto de Salud Carlos III, Majadahonda, Madrid, Spain; f Departamento de Bioquímica y Biología Molecular, Universidad Complutense de Madrid, Madrid, Spain; Texas A&M University

**Keywords:** viral quasispecies, genome diversification, NS5A-NS5B amplicons, haplotype frequency, SOM network, prototype vector, fitness platform

## Abstract

RNA viruses replicate as complex mutant spectra termed viral quasispecies. The frequency of each individual genome in a mutant spectrum depends on its rate of generation and its relative fitness in the replicating population ensemble. The advent of deep sequencing methodologies allows for the first-time quantification of haplotype abundances within mutant spectra. There is no information on the haplotype profile of the resident genomes and how the landscape evolves when a virus replicates in a controlled cell culture environment. Here, we report the construction of intramutant spectrum haplotype landscapes of three amplicons of the NS5A-NS5B coding region of hepatitis C virus (HCV). Two-dimensional (2D) neural networks were constructed for 44 related HCV populations derived from a common clonal ancestor that was passaged up to 210 times in human hepatoma Huh-7.5 cells in the absence of external selective pressures. The haplotype profiles consisted of an extended dense basal platform, from which a lower number of protruding higher peaks emerged. As HCV increased its adaptation to the cells, the number of haplotype peaks within each mutant spectrum expanded, and their distribution shifted in the 2D network. The results show that extensive HCV replication in a monotonous cell culture environment does not limit HCV exploration of sequence space through haplotype peak movements. The landscapes reflect dynamic variation in the intramutant spectrum haplotype profile and may serve as a reference to interpret the modifications produced by external selective pressures or to compare with the landscapes of mutant spectra in complex *in vivo* environments.

**IMPORTANCE** The study provides for the first time the haplotype profile and its variation in the course of virus adaptation to a cell culture environment in the absence of external selective constraints. The deep sequencing-based self-organized maps document a two-layer haplotype distribution with an ample basal platform and a lower number of protruding peaks. The results suggest an inferred intramutant spectrum fitness landscape structure that offers potential benefits for virus resilience to mutational inputs.

## INTRODUCTION

High viral mutation rates lead to the generation of complex and dynamic mutant spectra termed viral quasispecies, which are important for adaptability to changing environments (1). In the case of hepatitis C virus (HCV), quasispecies complexity in infected patients (quantified as the number of different genomes estimated to be present in the replicating mutant ensembles as sampled from serum and liver samples) can exert an influence on disease progression and response to antiviral treatment (2–4) (reviewed in references 5 and 6). An understanding of the mechanisms that modify mutant distributions *in vivo* can be facilitated by minimizing the number of selective constraints during viral replication. This can be approached with cell culture systems that sustain long-term virus replication, as is the case of HCV replicating in Huh-7.5 cells (7–10).

The objective of the present work has been to approximate an inferred fitness landscape from the haplotype composition within individual mutant spectra of sequential HCV populations sampled while they replicated in a noncoevolving cellular environment. No external selective constraints were applied, and only perturbations inherent to the cell culture and the changing mutant spectrum of the replicating virus were present (11). Here, we report the haplotype relatedness and frequencies of an initial clonal HCV population and several of its derivatives resulting from up to 210 serial passages in human hepatoma Huh-7.5 cells (equivalent to about 730 days of continuous replication) (11–14). The starting clonal HCV population, termed HCV p0, was generated by transcription of plasmid Jc1FLAG2(p7-nsGluc2A) (15), followed by RNA electroporation into Huh-Lunet cells and minimum amplification of the progeny virus in Huh-7.5 cells (12). In this experimental design, fresh cells were infected with the virus shed into the cell culture medium of the previous infection so that cellular evolution was prevented. Each passage involved infection of 4 × 10^5^ Huh-7.5 reporter cells with 4 × 10^4^ to 4 × 10^6^ HCV 50% tissue culture infective dose (TCID_50_) units (depending on the passage number) (11, 13). The multiplicity of infection (MOI) was 0.1 to 10 TCID_50_/cell. Under these conditions, possible distorting effects of stochasticity on the evolution of quasispecies composition due to bottleneck effects in the course of virus passaging should be limited (16). Also, accumulation of defective genomes was largely avoided, as suggested by the constancy of specific infectivity along the 200 virus passages and the fact that biological and molecular clones retrieved from the passaged virus displayed similar sequence diversification (11).

**FIG 1:**
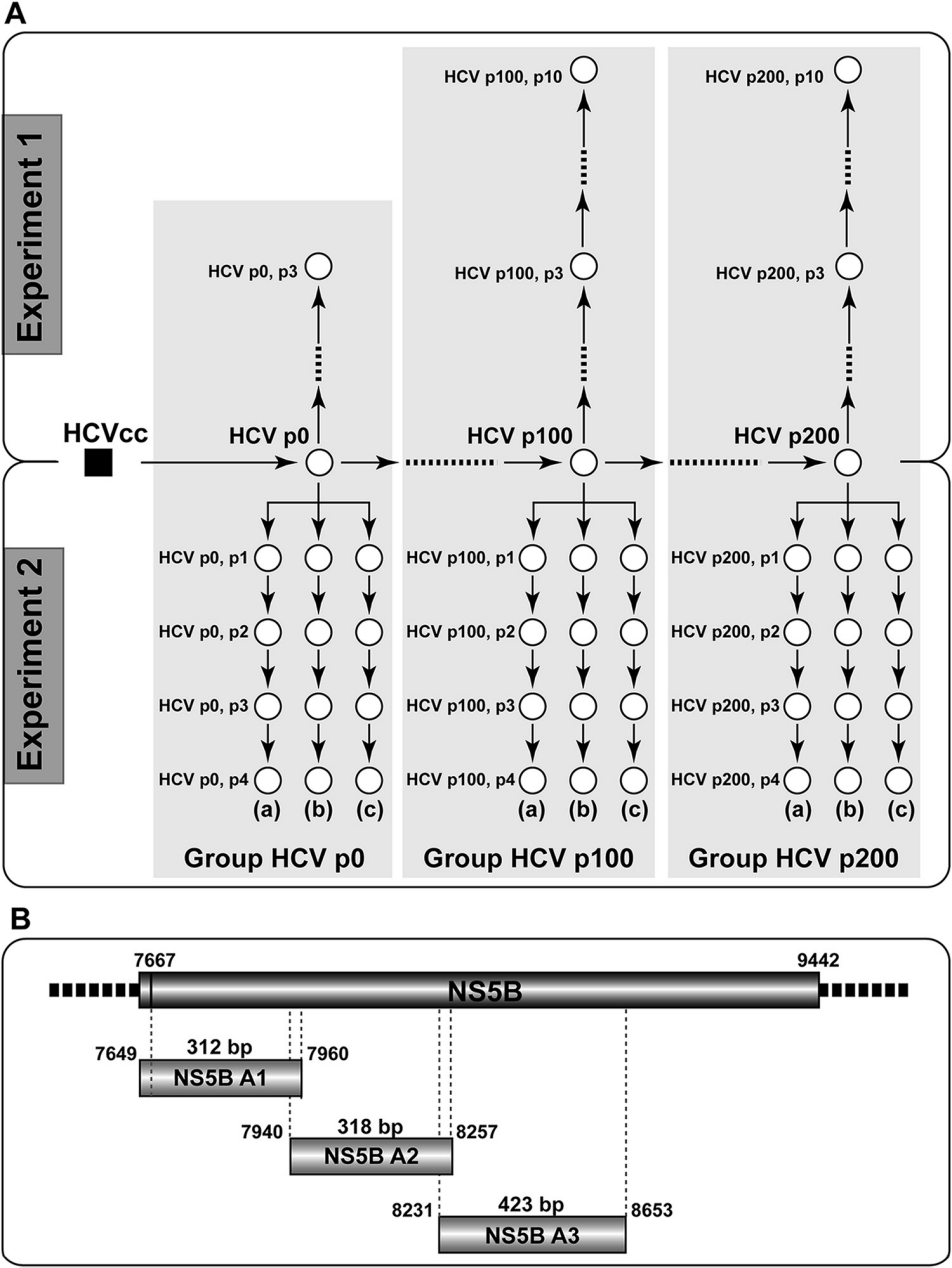
Experimental design and HCV amplicon analysis. (A) Schematic representation of the passages underwent by HCV p0 (derived from HCVcc (initial cell culture HCV population) [12]; Materials and Methods) in Huh-7.5 reporter cells. Populations are depicted as empty circles, and passage number is indicated by p (HCV p100, p3 means population HCV p100 subjected to three passages in Huh-7.5 cells). Experiment 1 (upper part) and experiment 2 (lower part) were performed starting with samples of the same HCV p0, HCV p100, and HCV p200 populations. In experiment 2, (a), (b), and (c) indicate triplicate passage series performed in parallel. A total of 44 HCV populations (corresponding to the empty circles) were analyzed by deep sequencing; Groups HCV p0, HCV p100, and HCV p200 (shaded in gray) gather the ensemble of populations more closely related to populations HCV p0, HCV p100, and HCV p200, respectively. The mutations (and deduced amino acid substitutions) identified in the populations from experiment 1 were reported in reference 18 and those in the populations from experiment 2 in reference 11. (B) HCV genomic residues 7,649 (NS5A-coding region) to 8,653 (NS5B-coding region) (genome numbering according to reference isolate JFH-1) and length in base pairs (bp) of amplicons A1, A2, and A3 analyzed by Illumina MiSeq sequencing. Note that the 21 most 3′-terminal nucleotides of A1 are redundant with the 21 most 5′-terminal nucleotides of A2 and that the 27 most 3′-terminal nucleotides of A2 are redundant with the most 5′-terminal nucleotides of A3. Further details on virus origin, GenBank accession numbers, and sequencing procedures are given in Materials and Methods.

Our previous comparative analyses of HCV p0 and the derived populations at passages 100 and 200 (termed HCV p100 and HCV p200, respectively) revealed several phenotypic modifications, concomitantly with the process of adaptation to cell culture. The modifications included enhanced resistance to antiviral agents in the absence of specific inhibitor escape amino acid substitutions (12, 17–21) as well as increases in virus particle density, in capacity to kill host cells, and in the extent of shutoff of host cell protein synthesis (13). Both, HCV p100 and HCV p200 exhibited a 2.3-fold increase in replicative fitness relative to the initial population HCV p0 arbitrarily assigned a fitness of 1.0, as measured by growth-competition experiments in Huh-7.5 cells (13, 17). This procedure constitutes a dynamic fitness measurement because it is carried out while the populations replicate. The reason why the dynamic fitness was similar at passages 100 and 200 may lie in increasing difficulty to incorporate the constellations of mutations required for fitness gain or to viral population size limitations, as previously documented with vesicular stomatitis virus (22, 23). However, not all replicative parameters (calculated for each population individually) plateaued at passage 100. In a five-serial-passage test in Huh-7.5 cells, the intracellular exponential growth rate was 17- and 45-fold larger for HCV p100 and HCV p200, respectively, than for HCV p0 (13). In contrast, the maximum extracellular infectious progeny attained was 1.17-fold higher for both HCV p100 and HCV p200 than for HCV p0, in agreement with the dynamic fitness values denoted by the competition experiments (13). HCV p0, HCV p100, and HCV p200 were subjected to additional passages in Huh-7.5 cells (Fig. 1A) under the standard infection conditions, as described in Materials and Methods.

Deep sequencing of the genome of populations that were sampled to monitor the evolution from HCV p0 to HCV p200 revealed a large number of mutations that varied in frequency even between successive passages; we referred to the effect of these types of mutations as mutational waves (13). Strikingly, the waves did not subside when the population had increased its adaptation to the cellular environment, and they were even more pronounced at late than at early viral passages (11, 24, 25), suggesting absence of a population equilibrium (in the sense of a steady distribution of variant genomes). This seemingly paradoxical observation begged for an examination of the possible modifications underwent by the fitness landscape of individual HCV populations (inferred from the intramutant spectrum distribution frequency of individual sequences) in their transition from HCV p0 to HCV p200. What is inferred can be defined as a static fitness landscape because it is based on the haplotype distribution at a fixed time point (passage number) of the population under study.

Previous studies (derived generally from comparison of consensus sequences or of individual clones separated from their mutant spectrum context) have afforded evidence that fitness landscapes for RNA viruses replicating in their natural environments are rugged and variable (16, 26–33). Fitness effects of mutations or amino acid substitutions have often been inferred from predicted or experimentally verified activity or stability of virus-coded proteins or from the replicative performance of reconstructed viruses (34–39). An alternative approach has been to derive fitness landscapes from mutation frequencies calculated either from standard (consensus) sequences or from ultradeep sequencing (UDS) data (27, 31, 38, 40).

No studies have compared intramutant spectrum haplotype landscapes of sequential viral populations and landscape modifications following extensive viral replication in cell culture in the absence of external selective constraints, as is the case of the evolution from HCV p0 to HCV p200. To this aim, we have applied an artificial neural network (ANN) procedure as a learning method to derive self-organized maps (SOMs) (41, 42). This procedure has an advantage over standard methods of multidimensional scaling in that it permits establishing connections among the points visualized in the data set (see Materials and Methods). Indeed, the Kohonen’s SOM algorithm classifies a set of input data vectors (in our case viral genomic sequences) in a two-dimensional (2D) map. By an unsupervised process, it groups data vectors by relatedness, projecting those vectors that have similar content in neighboring regions of the map (2D grid). In this manner, an ordered grid is generated in which each node (neuron) is associated with a reference RNA sequence (30, 43). Each neuron of the network maps all input sequences that fall within a distance from its reference vector, which is smaller than the distance to the rest of reference vectors. Because vectors represent viral genomic sequences, to calculate numerical distances between vectors, a codification algorithm has been used, as previously described (43) (details are given in Materials and Methods and Fig. S1 in https://saco.csic.es/index.php/s/sFQnRii4dC94LRN). The SOM procedure brings together two functions: (i) vector quantification (clustering) and (ii) vector projection, where the high-dimensional input space (determined by the length in base pairs of the haplotypes) is projected onto the 2D output layer of the network. Furthermore, the SOM algorithm maps the training sequences with low genetic distance in nearby neurons on the 2D grid, while distant sequences are mapped in distant neurons; that is, the distance among neurons in the 2D grid preserves the genetic distance of the training sequences. The third dimension (3D) of the haplotype map (*z* axis) was raised from the 2D SOM grid by using for each neuron the percentage of sequences (UDS reads) that mapped in that neuron (in the context of haplotype map construction, the terms sequences and UDS reads are used indistinctly throughout the manuscript; UDS reads are the clean reads obtained from the Illumina MiSeq platform, as described in Materials and Methods).

The SOM analysis of 44 HCV mutant spectra from populations derived from HCV p0, HCV p100, and HCV p200 has disentangled the modifications of the intramutant spectrum haplotype landscape during long-term adaptation of HCV to Huh-7.5 cells. The SOM display suggests a remarkable intrapopulation topology consisting of a discrete number of high-frequency haplotypes emerging from a lower frequency layer that approximates a platform. The landscape is modified in a highly dynamic way following virus passage, as evidenced by an almost complete shift in the region of sequence space occupied by the analyzed amplicons during the last 100 serial infections. Implications of the two-level haplotype topology in terms of the inferred intramutant spectrum static fitness are discussed.

## RESULTS

### Self-organized maps and haplotype landscape of mutant spectra of HCV populations.

A clonal HCV p0 population derived from plasmid Jc1FLAG2(p7-nsGluc2A) (12, 15) was subjected to 200 serial passages in Huh-7.5 reporter cells. Samples from the initial population and from HCV p100 and HCV p200 were further passaged up to 10 times in two separate experiments and several replicas, thus providing a total of 44 HCV populations for UDS analysis (populations are depicted as empty circles in Fig. 1A). Three amplicons (termed A1, A2, and A3), extending from HCV genome residues 7,649 to 8,653 (residue numbering according to isolate JFH-1, accession number AB047639), were analyzed (Fig. 1B). Amplicon A1 spans residues 7,649 to 7,960 (which correspond to amino acids 461 of NS5A to amino acid 98 of NS5B). A2 spans residues 7,940 to 8,257 (amino acids 92 to 197 of NS5B), and A3 covers residues 8,231 to 8,653 (amino acids 189 to 329 of NS5B). The number of processed, clean reads and the deduced number of haplotypes (number of identical reads represented by a nucleotide sequence) for the three amplicons are given in Table 1. In our sequence analysis and processing, no insertion-deletions (indels) were recorded; their exclusion is justified by our evidence that they may arise artifactually in homopolymeric tracts upon RNA amplification (13).

**TABLE 1:** Number of reads and haplotypes derived from MiSeq Illumina sequencing of HCV amplicons A1, A2, and A3

			No. of reads (no. of haplotypes^*a*^)		
Expt	Virus	Passage	Amplicon 1 (7,649 to 7,960)^*b*^	Amplicon 2 (7,940 to 8,257)^*b*^	Amplicon 3 (8,231 to 8,653)^*b*^
Expt 1	HCV p0	Initial	240,376 (8)	189,190 (4)	122,836 (3)
		p3	243,755 (6)	273,783 (2)	119,705 (4)
	HCV p100	Initial	225,977 (18)	251,949 (10)	108,548 (14)
		p3	201,355 (14)	282,139 (10)	87,596 (15)
		p10	188,215 (8)	197,078 (7)	79,412 (9)
	HCV p200	Initial	50,502 (15)	166,060 (11)	50,111 (11)
		p3	53,462 (13)	160,179 (10)	62,872 (12)
		p10	57,730 (16)	149,758 (12)	51,910 (10)
Expt 2a	HCV p0	p1	18,817 (4)	45,378 (4)	6,759 (3)
		p2	25,866 (4)	61,599 (3)	9,693 (3)
		p3	33,964 (5)	112,247 (4)	6,690 (4)
		p4	46,180 (3)	159,699 (3)	6,695 (5)
	HCV p100	p1	32,729 (15)	119,433 (5)	6,698 (15)
		p2	34,670 (14)	138,215 (5)	8,040 (15)
		p3	29,787 (16)	135,219 (5)	7,621 (17)
		p4	35,007 (13)	115,195 (5)	7,101 (16)
	HCV p200	p1	24,630 (13)	52,247 (15)	8,862 (18)
		p2	31,553 (19)	136,972 (13)	8,917 (14)
		p3	32,166 (20)	102,391 (13)	7,137 (15)
		p4	54,605 (19)	179,432 (15)	13,687 (14)
Expt 2b	HCV p0	p1	148,212 (9)	149,731 (2)	64,912 (2)
		p2	195,318 (5)	137,365 (3)	74,195 (3)
		p3	138,832 (6)	120,688 (4)	60,089 (4)
		p4	122,525 (4)	177,166 (5)	83,941 (4)
	HCV p100	p1	128,168 (15)	149,817 (8)	52,351 (15)
		p2	116,120 (16)	84,408 (6)	75,494 (17)
		p3	120,016 (17)	117,116 (5)	53,153 (17)
		p4	117,114 (16)	120,914 (6)	85,711 (17)
	HCV p200	p1	155,347 (17)	124,204 (15)	53,455 (16)
		p2	152,250 (18)	116,427 (14)	62,190 (11)
		p3	122,481 (12)	95,088 (14)	44,088 (13)
		p4	85,906 (19)	135,373 (14)	73,119 (13)
Expt 2c	HCV p0	p1	104,149 (8)	90,216 (3)	134,903 (2)
		p2	209,396 (8)	130,509 (3)	54,018 (2)
		p3	86,558 (5)	69,588 (3)	124,363 (3)
		p4	155,428 (5)	144,968 (6)	44,764 (3)
	HCV p100	p1	73,080 (18)	72,919 (9)	123,388 (16)
		p2	186,831 (17)	79,384 (8)	55,436 (18)
		p3	98,930 (18)	75,155 (6)	125,049 (18)
		p4	137,944 (17)	115,719 (7)	61,177 (18)
	HCV p200	p1	85,384 (20)	106,672 (16)	117,945 (14)
		p2	141,307 (17)	107,340 (16)	75,365 (14)
		p3	106,261 (21)	138,379 (12)	94,545 (15)
		p4	141,033 (21)	159,476 (17)	58,184 (15)

a The experiments, HCV populations, and amplicon numbers are those described in Fig. 1. Mutations were counted relative to the HCV sequence encoded in plasmid Jc1FLAG2(p7-nsGluc2A), as previously described (11, 18). The total number of reads and haplotypes (in parenthesis) were derived as detailed in Materials and Methods.

b The HCV genomic residues spanned by each amplicon are 7,649 to 7,960 (amplicon 1), 7,940 to 8,257 (amplicon 2), and 8,231 to 8,653 (amplicon 3) (numbering according to isolate JFH-1, accession number AB047639).

For each of the 44 viral populations and each amplicon, an individual haplotype map was constructed following five steps (see Materials and Methods for additional information on SOM derivation). (i) A FASTA file with the haplotype sequences, including the corresponding HCV genomic sequence contained in plasmid Jc1FLAG2(p7-nsGluc2A) (which is also used as reference for mutation counting), was prepared (Fig. S2 in https://saco.csic.es/index.php/s/sFQnRii4dC94LRN). (ii) The sequence of each haplotype was labeled with a name, the number of identical sequences that define it, and its frequency (Fig. S3 in https://saco.csic.es/index.php/s/sFQnRii4dC94LRN). (iii) The 3D irregular codification (43) was used to transform nucleotide sequences into numerical vectors. In this procedure, each nucleotide is located at a vertex of an irregular tetrahedron with distance 1 between A-G and C-U vertices and distance 2 between the rest of pairs; that is, a distinction was made among mutation types. (iv) The codified sequences in each amplicon were used to train a SOM that comprised a set of neurons, each with a prototype vector, organized in a 15 × 15 2D neuron grid; a different SOM was trained for each amplicon using the 44 HCV populations (i.e., 572 haplotypes for amplicon A1, 358 haplotypes for amplicon A2, and 487 haplotypes for amplicon A3) (Table 1). In this way, training sequences were mapped around the neuron with the prototype vector that best matched in terms of Euclidean distance (the “best matching” unit or bmu [41, 42]). (v) On the 2D neuron grid, the third dimension (3D) was given by the frequency of each group of sequences mapped around each neuron, using for this calculation the sum of frequencies of the haplotypes mapped in the corresponding neuron. Although each SOM was trained with all the haplotypes of the amplicon, to raise the 3D map, only the subset of haplotypes of the HCV population of interest was used. Thus, in each 3D graph, the sum of heights considering all peaks is 100 so that the map displays the real measure of the distribution of individual sequences per neuron. With this quantified third (*z* axis) dimension, the 2D grid unfolded into a three-dimensional (3D) haplotype map. The 15 × 15 2D grids with haplotype identification of each neuron and 3D maps are compiled in Fig. S4 to S9 in https://saco.csic.es/index.php/s/sFQnRii4dC94LRN, with additional numerical information in links quoted therein.

**FIG 2:**
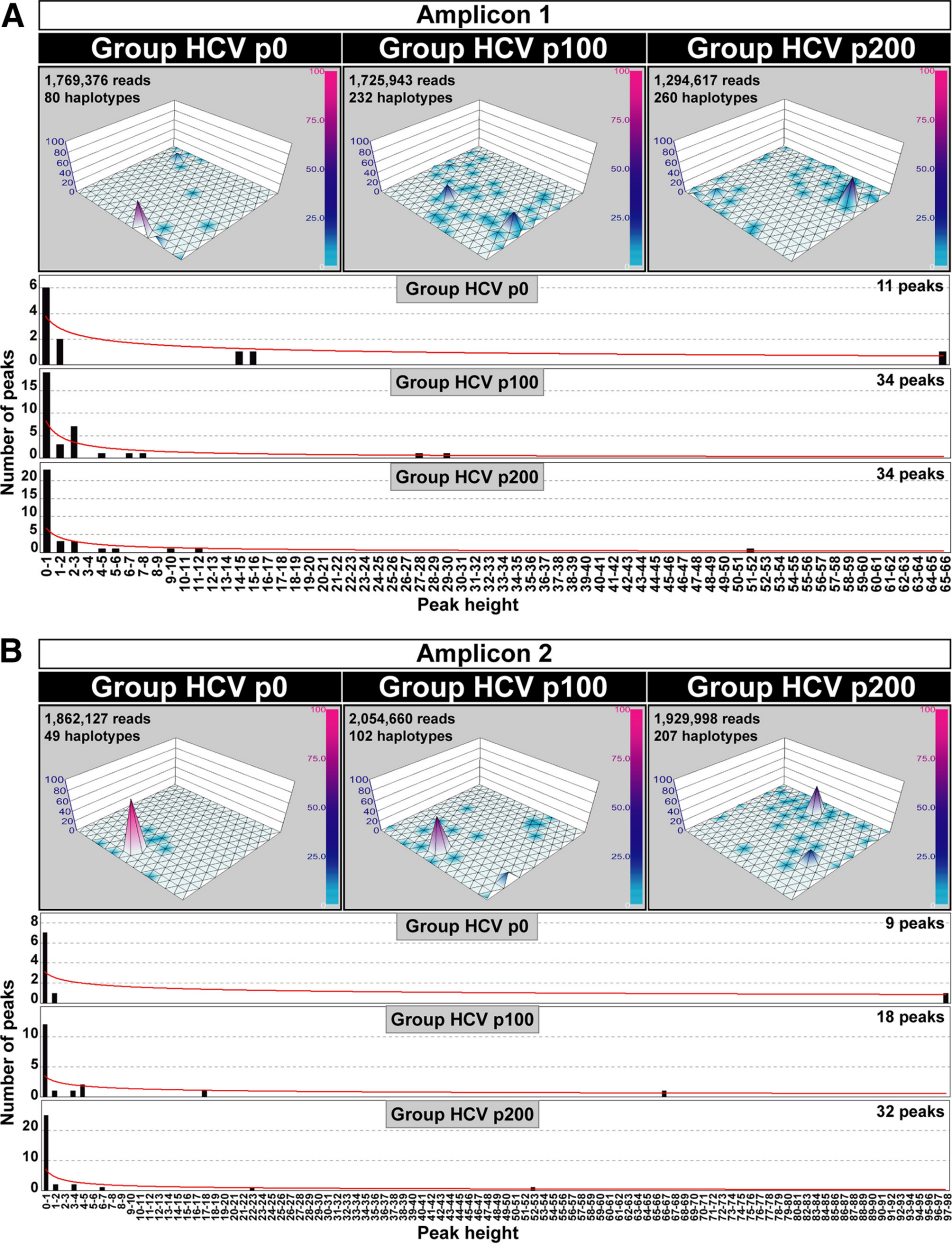

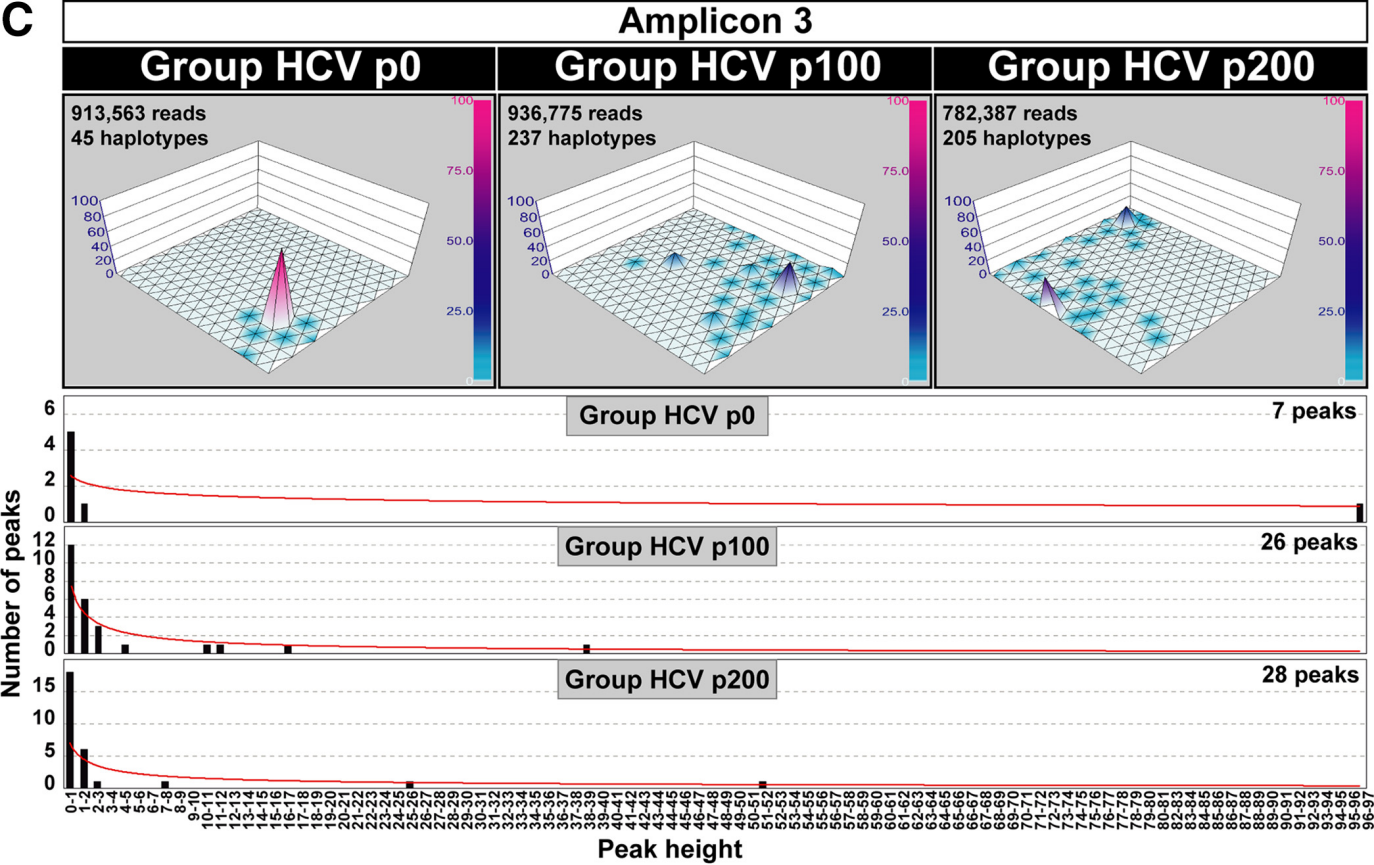
SOM-derived haplotype maps and number of peaks distributed according to haplotype abundance. The 15 × 15 neuron grids are displayed for Group HCV p0, Group HCV p100, or Group HCV p200 (defined in Fig. 1A). The number of sequences, haplotypes, and peaks is indicated inside each panel. Peak height (*z* axis) is determined by haplotype abundance, quantified from UDS reads. Normalized peak heights have been visualized with a color code with a scale included at the right of each haplotype graph. The distribution of number of haplotype peaks (ordinate) versus peak height (sequence abundance in unit range given in abscissa) is displayed below the haplotype maps of each amplicon. The relationship is described by the following functions: (A) amplicon 1: Group HCV p0, *y* = 3.7934*x*^−0.403^ (*R*^2^ = 0.7672); Group HCV p100, *y* = 8.2657*x*^−0.77^ (*R*^2^ = 0.6463); Group HCV p200, *y* = 6.6996*x*^−0.709^ (*R*^2^ = 0.5974); amplicon 2: Group HCV p0, *y* = 3.1334*x*^−0.281^ (*R*^2^ = 0.3804); Group HCV p100, *y* = 3.4527*x*^−0.395^ (*R*^2^ = 0.3649); Group HCV p200, *y* = 7.0358*x*^−0.638^ (*R*^2^ = 0.5818); (B) amplicon 3: Group HCV p0, *y* = 2.5728*x*^−0.233^ (*R*^2^ = 0.3807); Group HCV p100, *y* = 7.453*x*^−0.723^ (*R*^2^ = 0.7755); Group HCV p200, *y* = 6.97*x*^−0.629^ (*R*^2^ = 0.5886). The origin of the sequences, derived haplotypes, and procedures are described in Materials and Methods.

Because no major differences in haplotype maps were observed between the populations passaged in experiments 1 and 2, and among parallel passage replicas (the HCV populations and their connections are depicted in Fig. 1A), composite maps that included all the populations derived from HCV p0, those derived from HCV p100, or those derived from HCV p200 were obtained separately for each amplicon. The composite haplotype maps allow a more robust comparative examination of three groups of HCV populations, consisting of HCV p0, HCV p100, or HCV p200, each together with their direct derivatives, which are separated by a maximum of 10 serial passages. The three groups are named Group HCV p0, Group HCV p100, and Group HCV p200, respectively (Fig. 1A). This is the nomenclature used throughout the manuscript to distinguish the groups from their initial single populations HCV p0, HCV p100, and HCV p200. The comparison of the haplotype landscape for the three groups reveals an expansion of the total number of haplotype peaks in the evolution from Group HCV p0 to either Group HCV p100 or Group HCV p200 (fold increase range of 2.1 to 5.3 for the number of haplotypes and of 2.0 to 4.0 for haplotype peaks) (the number of sequences, haplotypes, and haplotype peaks is given inside each panel of Fig. 2). The increase of the number of different haplotypes was significant in the evolution from Group HCV p0 to Group HCV p100 (*P* = 0.0439; *t* test) and from Group HCV p0 to Group HCV p200 (*P* = 0.0015; *t* test), while the difference between Group HCV p100 and Group HCV p200 did not reach statistical significance (*P* = 0.2687; *t* test). Likewise, the difference in the number of haplotype peaks between Group HCV p0 and Group HCV p100 and between Group HCV p0 and Group HCV p200 was statistically significant (*P* = 0.0295 and *P* = 0.0004, respectively; *t* test), while the difference between Group HCV p100 and Group HCV p200 did not reach statistical significance (*P* = 0.1857; *t* test) (*t* test values and confidence intervals for these differences are given in Table S1 in https://saco.csic.es/index.php/s/sFQnRii4dC94LRN).

The distribution of the number of peaks as a function of peak height was similar for the three amplicons (*P* = 1; chi-square test). In all cases, there is an accumulation of the number of haplotype peaks within the peak height range 0 to 1 (graphics in Fig. 2). Considering the three amplicons together, the number of peaks in range 0 to 1,1 to 2, and all other range values was 18, 4, and 5, respectively, for Group HCV p0; the corresponding values were 43, 10, and 25 for Group HCV p100 and 66, 11, and 17 for Group HCV p200 (data in Fig. 2). The abundance of the number of peaks that fall within the 0 to 1 height range is also evidenced by the average ratio for all amplicons and populations of 0.66 (range 0.46 to 0.78) between the number of haplotype peaks within the 0 to 1 height range and the number of peaks that fall into any other height range. The dominance of low-height peaks is also recapitulated in the function that relates the number of peaks with their sequence abundance at each neuron (third dimension in the haplotype plot) (the graphics are shown below the maps depicted in Fig. 2, and the equations are given in the figure legend).

Shifts in the occupation of sequence space (position of peaks in the 2D grid) were observed for the three amplicons (Fig. 2). The position of the haplotype peaks moved in their location in the three population groups, except for the most prominent peak of amplicon 2 in Group HCV p0 that was also present in Group HCV p100. This shared peak was represented by a haplotype of identical sequence in each of the HCV p0 and HCV p100 populations that was integrated into the composite maps; the sequence was coincident with that present in the parental plasmid Jc1FLAG2(p7-nsGluc2A) (Table S2 in https://saco.csic.es/index.php/s/sFQnRii4dC94LRN). The consistency of peak display among replicas of the same population (data given in Fig. S7 to S9 in https://saco.csic.es/index.php/s/sFQnRii4dC94LRN) validates the differences observed among the three groups. Therefore, the replicative fitness increase in the evolution from HCV p0 to either HCV p100 or HCV p200 (13) was reflected mainly in the number of low haplotype peaks that occupied an increased, albeit shifting, portion of sequence space.

### Shared and unique haplotype peaks among HCV populations.

**FIG 3:**
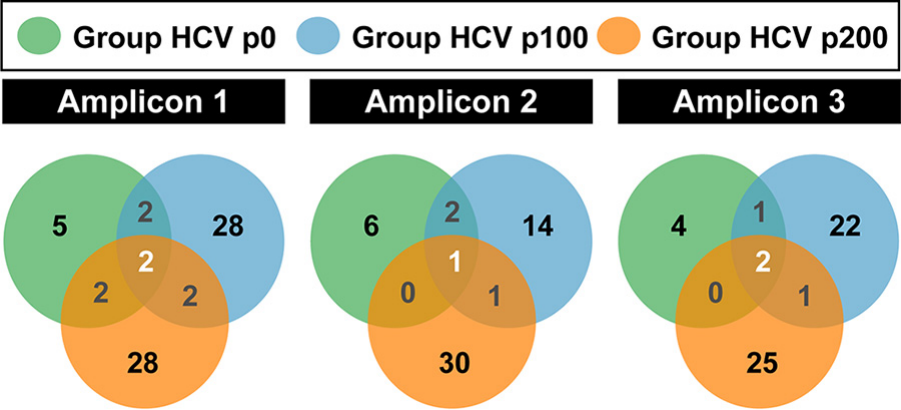
Distribution of haplotype peaks among HCV groups. Venn diagrams indicating for each amplicon the number of peaks unique to one HCV group and those shared by two or more HCV groups. Populations are color coded. Peak identity was determined according to data summarized in Table 1, Fig. 2, and in the supplemental material; statistical significances are given in Table S3 (https://saco.csic.es/index.php/s/sFQnRii4dC94LRN).

To express quantitatively the spread of mutant spectra in sequence space upon evolution from HCV p0 to HCV p100 and HCV p200, the number of peaks unique to one of the groups (HCV p0, HCV p100, or HCV p200) and the number of peaks shared by two or three groups was recorded (Fig. 3 and Table S2 in https://saco.csic.es/index.php/s/sFQnRii4dC94LRN). The ratio of number of unique peaks in Group HCV p0 relative to the number of peaks shared by the three groups was 2.5, 6, and 2 for amplicons A1, A2, and A3, respectively; the corresponding ratios were 14, 14, and 11 for Group HCV p100 and 14, 30, and 12.5 for Group HCV p200. The values were similar when the ratio was calculated relative to the number of peaks shared with any of the other groups; in this case, the ratios for Group HCV p0 were 2.5, 3, and 4 for amplicons A1, A2, and A3, respectively, and increased to 14, 7, and 22, respectively, for Group HCV p100 and to 14, 30, and 25, respectively, for Group HCV p200. For Group HCV p0, the difference between the number of unique and shared peaks was not statistically significant (*P* = 0.50, *P* = 0.17, and *P* = 0.50 for amplicons A1, A2, and A3, respectively; proportion test). In contrast, for Groups HCV p100 and HCV p200, the bias in favor of unique versus shared peaks was highly significant. For Group HCV p100 the following *P* values were obtained: *P* = 1.76 × 10^−7^, *P* = 0.00135, and *P* = 1.209 × 10^−6^ for amplicons A1, A2, and A3, respectively (proportion test). For Group HCV p200, the following *P* values were obtained: *P* = 1.76 × 10^−7^, *P* = 7.392 × 10^−12^, and *P* = 9.972 × 10^−9^ for amplicons A1, A2, and A3, respectively (proportion test) (confidence intervals for these differences are given in Table S3 in https://saco.csic.es/index.php/s/sFQnRii4dC94LRN). Therefore, the diversification and progressive occupation of sequence space by clonal HCV upon replication in Huh-7.5 cells is confirmed by the number of haplotype peaks that are unique to Group HCV p100 and to Group HCV p200. The largest increase was scored by amplicon 2, in agreement with the quantification of the number of haplotypes and haplotype peaks (compare Fig. 2 and 3).

### Fused amplicons.

**FIG 4:**
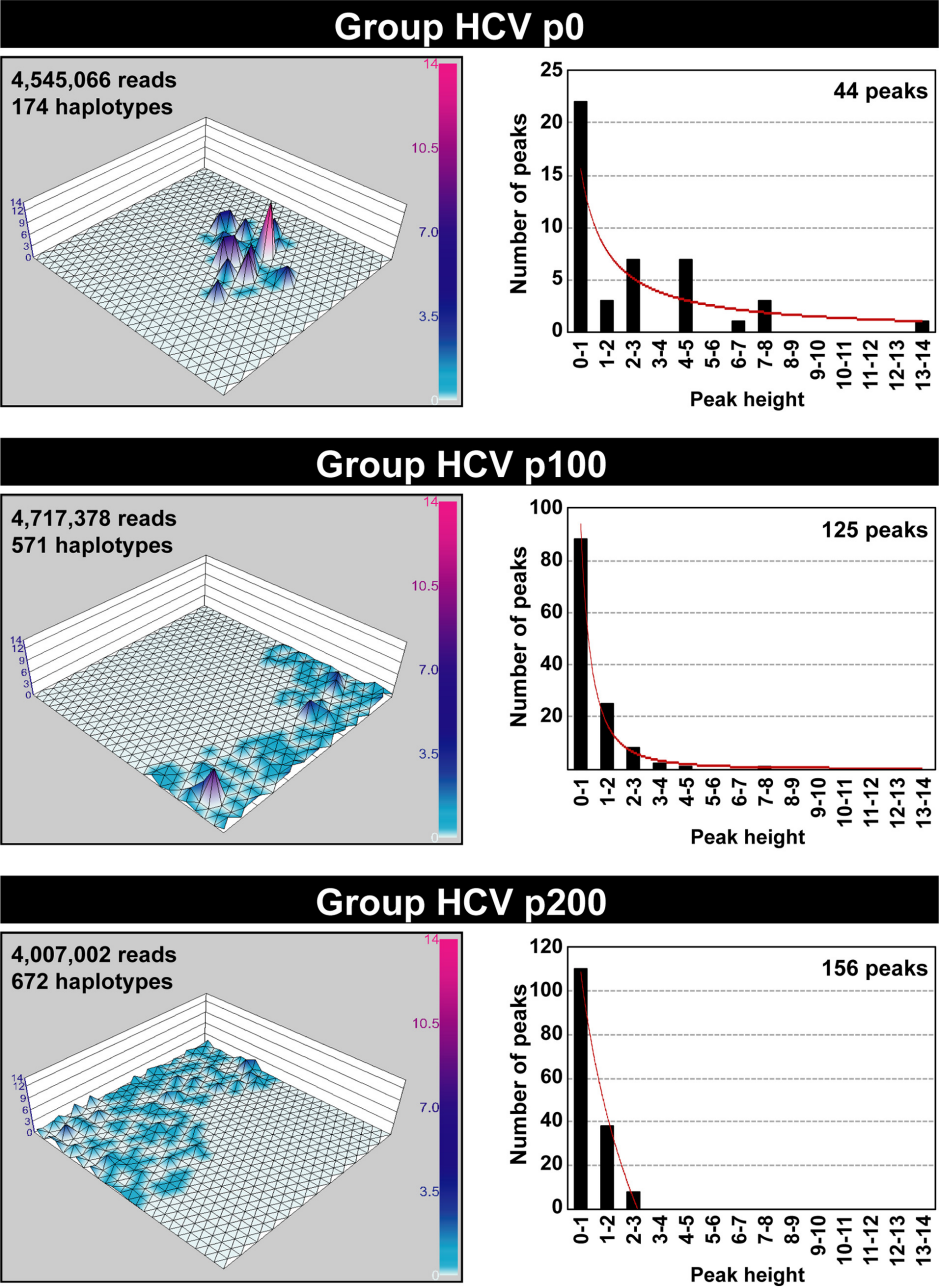
Haplotype maps constructed with the fused NS5B amplicons. The HCV group and number of sequences and haplotypes used for the 25 × 25 neuron graphic and the resulting number of haplotype peaks are indicated inside each map. Peak height is determined by sequence abundance, which is color coded with a scale included at the right of each map. The distribution of number of peaks (ordinate) versus peak height (sequence abundance in unit range displayed in abscissa) is indicated in the plots on the right. The relationship is described by the following functions: Group HCV p0, *y* = 15.659*x*^−1.001^ (*R*^2^ = 0.6519); Group HCV p100, *y* = 93.588*x*^2.441^ (*R*^2^ = 0.9931); Group HCV p200, −94.031ln(*x*) + 108.16 (*R*^2^ = 0.9931). Procedures are described in Materials and Methods.

To produce a global image of the haplotype landscape of the genomic region analyzed by incorporating the information of the three amplicons in a single graphic, it was necessary to equalize their length in nucleotides. Because the amplicons have overlapping sequences (Fig. 1B), we completed for each amplicon a length of 1,005 nucleotides using the missing sequence information provided by the other amplicons from the same population (procedure detailed in Materials and Methods). Then, a 25 × 25 Kohonen’s ANN was trained using the 1,417 resulting fused haplotypes (Table 1). As in the case of separate amplicons, haplotype maps were built for each of the 44 populations based on haplotype frequencies mapped at each neural unit. Because no major differences were noted among the individual maps, a composite landscape was recapitulated for Groups HCV p0, HCV p100, and HCV p200. The results (Fig. 4) show again the peak dispersion upon evolution from HCV p0 to HCV p100 and HCV p200 and renders evident a striking location displacement of peak abundance within the 2D grid. In the case of fused amplicons, the SOM training was carried out using the haplotypes that result from the fused sequences, which marks a difference with the procedure followed for the training of the three networks for the separate amplicons. Thus, by almost triplicating the genetic information contained in fused haplotypes with respect to the haplotypes of the individual amplicons, the genetic difference between the fused haplotypes of the groups was increased with respect to the genetic difference of the haplotypes from the individual amplicons. Using the fused haplotypes, it was possible to visualize more clearly on the grid the differences among the HCV quasispecies as the virus evolved over time (compare Fig. 2 and 4). In particular, peaks in Group HCV p100 and Group HCV p200 clumped at opposite grid localities. In conclusion, our data show that despite prolonged replication in a nonevolving cellular environment, the intramutant spectrum HCV haplotype landscape appears as remarkably broad, rugged, and dynamic, with a two layer peak height distribution.

### Mutation types and amino acid substitution tolerance in haplotypes from low and high haplotype peaks.

To analyze a possible difference in mutation types and amino acid substitution tolerance between sequences found in low and high haplotype peaks, the peaks were divided in two sets: one with the sequences that populate peaks of height range 0 to 1 and another set with sequences in peaks of height range 2 to 3 and higher (peak height distributions are given in Fig. 4). Using the HCV sequence in plasmid Jc1FLAG2(p7-nsGluc2A) as reference (15), the ratio of transition versus transversion mutations increased in a similar proportion for the haplotypes present in low and high peaks of Group HCV p100 and Group HCV p200. A similar increase was found for the ratio of synonymous versus nonsynonymous mutations (Fig. S10A and B and Tables S4 to S6 in https://saco.csic.es/index.php/s/sFQnRii4dC94LRN). Amino acid acceptability was determined with the PAM 250 matrix (44). The low acceptability substitution class (PAM 250 < 0) was less abundant in the haplotypes of the high peaks of Group HCV p200, but the difference with low haplotype peaks was not statistically significant (Fig. S10C in https://saco.csic.es/index.php/s/sFQnRii4dC94LRN). The comparisons of mutation types and amino acid substitution tolerance mark only tendencies in the diversification process. The dynamic fitness of the genomes whose mutations conform the haplotypes sampled in low or high peaks may be dictated by mutations located anywhere in the genome. The decrease of amino acid substitutions with class PAM 250 of <0 in haplotypes of the high peaks of Group HCV p200 may be due to negative selection acting on the genomes harboring them. Such a decrease is the only distinctive feature that we have identified in the mutation repertoire of the populations examined (compiled in Tables S4 to S6 in https://saco.csic.es/index.php/s/sFQnRii4dC94LRN).

## DISCUSSION

Genetic variability of RNA (and many DNA) viruses is a major feature of their biology and an obstacle for disease control. Numerous analyses of clinical and laboratory isolates have indicated that HCV is one of the most genetically variable RNA viral pathogens. Its plasticity results in considerable phenotypic heterogeneity that can influence disease progression and the effectiveness of antiviral interventions (reviews in references 5 and 6). Yet, the information on the number of different genomes and their relatedness within a mutant spectrum is very limited. Comparisons have been largely restricted to consensus genomic sequences from infected patients and have centered on the effect of antiviral interventions on viral population composition. In this line, an HCV sequence database was translated into an empirical fitness landscape to design vaccines that might simultaneously decrease viral fitness and avoid selection of escape mutants (45).

Our previous studies evidenced wide and dynamic diversification of mutant spectra in the evolution from the initial HCV p0 to HCV p100 and HCV p200 (11, 13, 18); haplotype alignments that are indicative of the diversification are available in https://saco.csic.es/index.php/s/586L2f9jJQtbRXq. As an interpretation of the results, we proposed broadly diversifying selection as an attribute of viral quasispecies dynamics, manifested when viruses replicate in environments that do not experience external perturbations (24). A likely driver of broadly diversifying selection is the modification of mutant spectrum composition due to mutational input, intrapopulation competition, selection, and drift (11, 24, 46). The diversity indices quantified in previous studies did not inform of the relationships among the sequences present within each mutant spectrum of the evolving HCV population. Such relationships have been approached in the present study with the ANN method SOM developed by Kohonen and colleagues (41, 42, 47). Our results (using UDS data to provide sequence and haplotype input) have evidenced that SOM is suitable to establish neighbor relationships among components of mutant clouds, which is essential to properly describe viral populations beyond the assignment of complexity indices (48). A major feature of the SOM method is that the distance among neurons in the 2D grid preserves the genetic distances among the training sequences. Such topology conservation is also the goal of multidimensional scaling algorithms. However, in contrast with the latter, SOM displays a 2D regular grid of interconnected neurons rather than individual haplotypes; this is an advantage when the data set is large. Additionally, SOM identifies clustered haplotypes in each neuron. Also, since the SOM map is shaped as a regular mesh, it can be directly raised into a 3D graphic with a compact surface or landscape, simply by assigning a numerical value to each neuron or node in the grid. The numerical value is given by the haplotype frequency calculated from the UDS reads. In multidimensional scaling algorithms, the generated data projection is a cloud of points (single sequences) without connection among them. Although it is possible to subsequently produce a graph that relates the data points in a basic mesh (49), the resulting structure has the shape of an irregular branched skeleton; to raise the structure into a 3D surface, it is necessary to perform an additional step (50). Thus, SOM unifies in a single method the ordering of haplotypes by similarity in a regular mesh that preserves the topology and facilitates the elevation of a third dimension without the need of additional methods of surface generation.

The SOM method has not been previously applied to rank internally the relative abundance and relatedness of the ensemble of genomes that populate a mutant spectrum, but it has been used to determine the fitness landscape of HIV-1 clones and populations (30). Other applications have included the interpretation of patterns of cellular gene expression (51, 52) or the analysis of taxonomic clustering of cellular and viral RNA sequences (43). In connection with HCV, SOM clustering was used to investigate hepatocellular carcinoma (HCC) development as the basis for tumor differentiation and invasiveness from expression levels of 12,600 genes in 50 HCC samples from patients with positive HCV serology (53). Also, Kohonen’s ANNs were trained to predict undiagnosed HCV infections and infection risk (54).

The SOM analysis of HCV groups has revealed a two-layer haplotype landscape. The first layer consists of multiple low peaks that tend to form a broad platform, covering multiple points in sequence space. This platform was already discernible in Group HCV p0, implying that it was initiated with the rounds of genome multiplication that occurred from the initial RNA transfection to the attainment of population HCV p0 (Fig. 1A) (12). In the evolution toward HCV p100 and HCV p200, the populations maintained the same pattern, with low peaks expanded toward larger areas of sequence space. The basal haplotype platform is adorned with a limited number of protruding peaks that resemble the standard representation of a rugged fitness landscape in the Wrightian sense (55).

Despite the similar landscape morphology, the haplotype maps of Groups HCV p0, HCV p100, and HCV p200 show differences in peak distribution, either considering individual amplicons or a fused single 2D SOM network that recapitulates the information from the three amplicons (Fig. 2 and 4). In particular, the comparison indicates dynamics of peak movements, with striking differences between Group HCV p100 and Group HCV p200 despite the core populations HCV p100 and HCV p200 having reached the same dynamic fitness value as measured by the standard growth-competition assays (13, 17). The only biochemical parameter that we identified (and that may fuel the dynamics of change from HCV p100 to HCV p200) is a 2.6-fold larger intracellular exponential growth displayed by HCV p200 relative to HCV p100 (13). However, the three NS5B amplicons did not follow the same trajectory of haplotype distribution modification. While for amplicons 1 and 3, the ratio of peaks or haplotypes unique to one group to those shared by other groups was the same for Group HCV p100 and Group HCV p200, for amplicon 2, it was two times higher for Group HCV p200 than for Group HCV p100 (derived from the graphics of Fig. 2 and 4 and included in Table 1).

In the landscapes for individual amplicons (Fig. 2) a few neurons map haplotypes present both in Group HCV p0 and Group HCV p100 and others that are present both in Group HCV p100 and Group HCV p200; these shared neurons include either identical or very closely related haplotypes. In some cases, the peak height at the shared neuron locations remains constant, while in other cases it varies among groups. This is indicative again of genome frequency modifications that may be the result of sampling in the course of virus passaging or of selective effects evoked by mutations arising anywhere in the viral genome. The picture obtained is that of a network of interconnected transient sequences that do not define linear evolutionary events. Despite the absence of sublineages with temporal continuity, the number of identical haplotype peaks that arose in independent passage replicas of the same starting population is remarkable (50.4% [range 37.5% to 72.7%] of the total for replicas [a], [b], and [c] of populations HCV p0, HCV p100, and HCV p200, subjected to four serial passages in Huh-7.5 cells). Similarity of behavior in separate evolutionary viral lineages suggests a component of determinism (predictability) in a system whose evolution should be strongly directed by stochastically arising mutations. This paradoxical behavior has been previously observed in different studies with other RNA viruses, and a number of possible underlying mechanisms have been proposed (56–60).

A more realistic perception of the complexity of the HCV haplotype landscape can be obtained by considering that the SOM maps have been constructed with haplotypes from amplicons that cover only 10% of the entire HCV genome. This is a limitation of our study, although achieving a similar depth of mutation detection for whole-genome amplicons as short amplicons is still technically challenging. A more populated basal platform than displayed in the SOM graphics of Fig. 2 and 4 is predicted if the analysis of haplotype frequencies were extended to additional genomic sites. The reason is that the sites of heterogeneity (defined as those with more than one nucleotide, as revealed by Sanger sequencing) were found along the entire genome of the same HCV populations (11).

The haplotype landscapes resulting from HCV replication in a monotonous environment can serve as a basis for comparison with the landscapes acquired by a mutant spectrum when a selective constraint is applied to the evolving population. In particular, the analysis should reveal if alternative mutational pathways are available to the virus to respond to a specific constraint. Also, how the mutant spectrum of the resident genomes is shaped in patients compared with cell culture may be informative of environmental complexity and adaptive mechanisms, and such a work is now in progress.

The three-dimensional haplotype maps we have derived can be used to infer an intramutant spectrum static fitness profile of the resident genomes at a given passage. This is because the genomes that populate a mutant spectrum are ranked according to their relative replicative fitness, in our case quantified by haplotype concentrations (16). The dynamic fitness of a complex viral population cannot be inferred from the dynamic fitness values determined experimentally with biological clones isolated from the population where they belong. The reason is that the dynamic fitness of individual biological clones is, on average, lower than that of the parental population from which they were isolated (61–63). The profiles we have obtained do not provide a dynamic fitness value of the type we obtained previously for populations HCV p100 and HCV p200 (13), and this is why we use the term static fitness. The profiles cannot identify the genomic sequences that are associated with high or low static fitness. The relevant conclusion we derive from the haplotype distributions is that HCV displays a shifting static fitness profile because the genomes that represent the high fitness subset in Group HCV p100 are different from the subset that represents the high fitness in Group HCV p200. The results are in line with increasing evidence of fitness landscapes of viruses being highly complex and dynamic, features that, according to our study, can be maintained even upon extensive replication in a noncoevolving cellular environment. In addition, the two-layer haplotype distribution revealed by our SOM analysis may have biological consequences. The first layer or platform may prevent mutations from driving genomes into low dynamic fitness pits. It may also act as a springboard for viral populations to reach higher dynamic fitness peaks. This should reduce the transition time between fitness peaks, which is a limitation of adaptability recognized in general evolutionary genetics (64–66).

## MATERIALS AND METHODS

### Origin of the HCV populations and serial passages in Huh-7.5 reporter cells.

The initial HCVcc population was obtained by *in vitro* transcription of plasmid Jc1FLAG2(p7-nsGluc2A) (15), followed by RNA electroporation into Huh-Lunet cells and further amplification in Huh-7.5 reporter cell monolayers to yield the parental population HCV p0 (12). HCV p0 was further passaged in Huh-7.5 reporter cells to obtain HCV p100 and HCV p200 (HCV p0 that were propagated 100 and 200 times in Huh-7.5 reporter cells, respectively), as has been previously described (13). The sequences to derive the SOM-based haplotype landscape were obtained from the three parental HCV p0, HCV p100, and HCV p200 populations subjected to further passages in Huh-7.5 cells and in two different experiments (experiment 1 and experiment 2) and several replicas (Fig. 1A). To initiate these passages, 4 × 10^5^ Huh-7.5 reporter cells were infected with HCV p0, HCV p100, or HCV p200 at a multiplicity of infection (MOI) of 0.03 TCID_50_/cell. For subsequent passages, 4 × 10^5^ fresh Huh-7.5 reporter cells were infected with the virus contained in 0.5 ml of the cell culture medium from the previous infection of the same lineage; the multiplicity of infection (MOI) ranged from 0.1 to 0.5 TCID_50_ per cell. In all passages, infections were allowed to proceed for 72 to 96 h. This yielded the 44 populations for which a haplotype landscape was determined (Fig. 1A). To facilitate comparisons, the sequences from populations that lie within 10 or less serial passages from HCV p0, HCV p100, or HCV p200 have been included in three groups, as delineated in Fig. 1A The sequences on which the present study is based have been previously reported (11, 18) and are available at https://saco.csic.es/index.php/s/586L2f9jJQtbRXq. To control for the absence of cross-contamination with virus from another population or replica, mock-infected cells were maintained in parallel with each infected culture, and each supernatant was titrated; no infectivity in the mock-infected cultures was detected in any of the experiments. Additional procedures, including titration of infectivity to determine TCID_50_ values and viral RNA quantification, have been previously described (11–13).

### RNA extraction, viral RNA amplification, and ultradeep HCV RNA sequencing.

Intracellular viral RNA was extracted from the initial HCV p0, HCV p100, and HCV p200 populations and their passaged derivatives using the Qiagen RNeasy kit (Qiagen, Valencia, CA, USA). HCV RNA was amplified by reverse transcription-PCR (RT-PCR) using Accuscript (Agilent) and specific HCV oligonucleotide primers that have been previously described (Table S10 in reference 11). Amplification products were analyzed by agarose gel electrophoresis using Gene Ruler 1 kb Plus DNA ladder (Thermo Scientific) as a molar mass standard. To ascertain absence of contaminating templates, all experiments included negative controls without template RNA. To avoid sequence representation biases as a consequence of redundant amplifications of the same initial RNA templates due to template molecule limitations, amplifications were carried out with template preparations diluted 1:10, 1:100, and 1:1,000. Only when at least the 1:100-diluted template produced a visible DNA band was molecular cloning performed using the DNA amplified from the undiluted template sample. PCR products were purified (QIAquick gel extraction kit, Qiagen), quantified (Pico Green assay), and tested for quality (Bioanalyzer DNA 1000, Agilent Technologies) before Illumina deep sequencing analysis (MiSeq platform with the 2 × 300-bp mode with v3 chemistry).

Several control experiments were performed in preparation of the ultradeep sequencing procedure to ensure the reliability of the mutations derived from clean reads for proper mutant spectrum characterization. They have been previously described (67–70) and they were as follows. First, we determined the basal error of the amplification and sequencing process using an infectious HCV cDNA clone to perform RT-PCR, the nested PCR, and ultradeep sequencing using Illumina MiSeq. Second, we quantified the PCR recombination frequency during the amplification steps using mixtures of wild type and a mutant clone to perform RT-PCR and the nested PCR and ultradeep sequenced using an Illumina MiSeq. Third, we ascertained the similarity of read composition in different RT-PCR amplifications and sequencing runs using different samples of the same RNA preparation. We concluded that mutations identified with a frequency above the 0.5% cutoff value and that were consistently found in the two DNA strands could be considered for the analyses. For additional details of the read cleaning procedures, criteria for mutation acceptance, and experimental controls with reconstructed HCV RNA mutant mixtures, see references 67–70.

### SOM derivation.

A detailed description of the SOM algorithm has been published elsewhere (43). The ANN model (41) exhibits an architecture consisting of a set of neurons arranged in a rectangular grid that defines a neighborhood relationship. The map size has been chosen to ensure sufficient dispersion of the sequences mapped in the grid while preserving the grouping of those that are similar; the resulting size is a function of the size of the data set. In the case of mapping each amplicon, a 15 × 15 grid was considered suitable, while for the map generated with the fused amplicons, the chosen size was 25 × 25 because of the greater number of sequences.

Every neuron has an associated prototype vector with the same nature and dimension as the input data set (in this work, the amplicon sequences). SOM generates a projection or mapping of the input space, usually high dimensional, in the two-dimensional topological structure of the network. The SOM training algorithm determines the way in which this mapping is created. This process iteratively modifies the SOM prototype vectors to fit them to the distribution of the input data space using a methodology similar to a regression. During the SOM training, each input vector is associated with the neuron that best matches with the pattern in any metric (the so-called “best matching unit” [bmu]). As a result of this process, the prototype vectors associated with the bmu and with all the neurons located in a neighborhood area around it are modified to move them closer to the input vector. In this work, the bmu has been calculated in terms of Euclidean distance. In the case of classification of vectors with sequence data, the algorithm requires a previous transformation into equivalent numerical vectors. This has been done using the previously described codification (30, 43). Each nucleotide is transformed into the corresponding 3D numerical coordinates in an irregular tetrahedron (Fig. S1 in https://saco.csic.es/index.php/s/sFQnRii4dC94LRN). In this way, each RNA sequence is transformed into a numerical vector of a dimension that is three times the length of the sequence, and this is the vector that is used by the SOM algorithm during the training process. After the training, SOM can determine similarities over the input vectors (amplicon sequences) in the sense that similar sequences will be mapped by the same neuron or by a neighboring neuron.

The random initialization of the synaptic vectors and the order of data presentation during the training process are the two stochastic factors that can influence the topology preservation of the SOM network. Therefore, we followed the recommendation to train several SOM networks with the same data set and to select the one with the highest precision (given by the lowest Kaski-Lagus error [*ξ_k_*_− 1_]) (71). The *ξ_k_*_− 1_ function combines the quantization error of each haplotype of the data set to the closest synaptic vector, with an index that measures the mapping continuity of the data set to the SOM grid. Thus, it captures the degree of robustness of the trained network. The following parameters have been used to train all networks: number of input neurons (*N*); dimension of the data set vectors (length of the sequence times 3); size of the output map 15 rows times 15 columns for the individual amplicon data sets and 25 rows times 25 columns for the fused amplicon data set; hexagon neighborhood connection (each neuron has six neighbors around it, including two at the top, two at the bottom, one on the left, and one on the right); initial neighborhood of 14 rows and 14 columns for the individual amplicon data sets and 24 rows and 24 columns for the fused amplicon data set; with neighborhood decrement at the end of each epoch (equivalent to the number of sequences in the data set); learning factor *α*(*t*) = *α*1 (1− *t*/*α*2), with *α*1 = 0.1 and *α*2 equal to the total iterations of the training algorithm; the total number of iterations is equal to the total number of epochs times the number of sequences. The total number of epochs is determined by the initial neighborhood + 5; that is, the algorithm carries out the necessary epochs so that the neighborhood area decreases until it affects only the bmu plus 5 additional fine-tuning epochs.

Finally, a labeling process was applied to each map. Using as a basis the network selected for each data set, the 3D haplotype map labeling was generated with the accumulated frequencies for each haplotype so that each neuron was assigned the sum of frequencies of the haplotypes for which it is the bmu. This value represents the cumulative frequency of sequences that fall in the Voronoi region of the neuron. Although the SOM map is generated or trained with all the sequences of each data set, the 3D maps can be obtained with the subset of sequences to be represented.

### Amplicon fusion method.

Based on the fact that the amplicons have overlapping sequences (Fig. 1B), we completed for each haplotype of an amplicon a length of 1,005 nucleotides using the nucleotide sequence provided by the haplotypes of the other two amplicons in the same population, passage number, and experiment. To achieve this for amplicon A1 (original length of 312 bases), the amplicon A2 haplotypes with initial overlapping sequences matching the last 21 bases of haplotype A1 were located. The same operation was conducted with the amplicon A3 haplotypes whose initial overlapping sequences matched the last 27 bases of any of the A2 haplotypes found in the previous step. Fusion sequences were obtained for the A2 and A3 haplotype lists. To generate the final fusion sequence, the bases of each position were compared, keeping the base for any position with identical nucleotides in all sequences or the IUPAC nucleotide ambiguity code associated with the combination of the bases when a position had more than one nucleotide. The 312 bases of the amplicon A1 haplotype were completed by adding the last 297 bases of the A2 fusion sequence followed by the last 396 bases of the A3 fusion sequence. A similar procedure was used to derive the amplicon A2 (original length of 318 bases) fusion sequence. The amplicon A1 haplotypes with final overlapping sequences matching the first 21 bases of haplotype A2 and the amplicon A3 haplotypes with initial overlapping sequences matching the last 27 bases of haplotype A2 were located. The fusion sequence was obtained for the A1 haplotype list and for the A3 haplotype list. The 318 bases of the amplicon A2 haplotype were completed, including the first 291 bases of the A1 fusion sequence at the beginning and the last 396 bases of the A3 fusion sequence at the end, as described to complete amplicon 1. Likewise, for amplicon A3 (original length of 423 bases), the amplicon A2 haplotypes with final overlapping sequences matching the first 27 bases of haplotype A3 and the amplicon A1 haplotypes with final overlapping sequences matching the initial 21 bases of any of the A2 haplotypes found in the previous step were located. The fusion sequence was obtained for the A1 haplotype list and for the A2 haplotype list. The 423 bases of the amplicon A3 haplotype were completed including at the beginning the first 291 bases of the A1 fusion sequence followed by the first 291 bases of the A2 fusion sequence. When no haplotypes with matching overlapping sequences were found in any of the other two amplicons, the full list of haplotypes of the mismatched amplicon was used to equalize the length. The number of haplotypes per fused amplicon is given in Results and in Table 1.

### Statistics.

The statistical significance of differences among the number of haplotypes and haplotype peaks of HCV p0, HCV p100, and HCV p200 was calculated with the *t* test because the data follow a normal distribution (*P* > 0.05; Shapiro-Wilk test). The differences between the distribution of haplotype peaks of HCV p0, HCV p100, and HCV p200 for each amplicon was calculated with the Pearson’s chi-square test. The comparison between the number of unique peaks and the number of shared peaks of HCV p0, HCV p100, and HCV p200 for each amplicon was calculated with a proportion test with the Yates continuity correction. All calculations were performed using software R version 4.0.2.

### Data availability.

The Illumina data have been deposited in the NCBI BioSample database under accession numbers SAMN18645452, SAMN18645453, SAMN18645456, SAMN18645457, SAMN18645460, SAMN18645463, SAMN18645464, and SAMN18645467 (BioProject accession number PRJNA720288) for experiment 1 and SAMN13531332 to SAMN13531367 (BioProject accession number PRJNA593382) for experiment 2. The haplotype alignments are available at https://saco.csic.es/index.php/s/586L2f9jJQtbRXq. The FASTA files are termed according to the experiment (Exp1, Exp2a, Exp2b, or Exp2c), population (HCV p0, HCV p100, or HCV p200), passage (initial, p1, p2, p3, p4, or p10), and amplicon (A1, A2, or A3).
